# Trichlorido(4,4′-dimethyl-2,2′-bipyridine-κ^2^ *N*,*N*′)(methanol-κ*O*)indium(III) methanol monosolvate

**DOI:** 10.1107/S1600536812035490

**Published:** 2012-08-23

**Authors:** Sadif A. Shirvan, Sara Haydari Dezfuli

**Affiliations:** a Department of Chemistry, Islamic Azad University, Omidieh Branch, Omidieh, Iran

## Abstract

In the title compound, [InCl_3_(C_12_H_12_N_2_)(CH_3_OH)]·CH_3_OH, the In^III^ atom is six-coordinated in a distorted octa­hedral geometry by two N atoms from a chelating 4,4′-dimethyl-2,2′-bipyridine ligand, one O atom from a methanol mol­ecule and three Cl atoms. In the crystal, inter­molecular O—H⋯O and O—H⋯Cl hydrogen bonds link the complex and solvent methanol mol­ecules. Intra­molecular C—H⋯Cl hydrogen bonds are also present in the complex.

## Related literature

For related structures, see: Abedi *et al.* (2012 ▶); Ahmadi *et al.* (2008 ▶); Alizadeh *et al.* (2010 ▶); Amani *et al.* (2009 ▶); Dong *et al.* (1987 ▶); Hojjat Kashani *et al.* (2008 ▶); Ilyuhin & Malyarick (1994 ▶); Kalateh, Ahmadi *et al.* (2008 ▶); Kalateh, Ebadi *et al.* (2008 ▶); Kalateh *et al.* (2010 ▶); Malyarick *et al.* (1992 ▶); Shirvan & Haydari Dezfuli (2011 ▶, 2012 ▶); Yousefi *et al.* (2008 ▶).
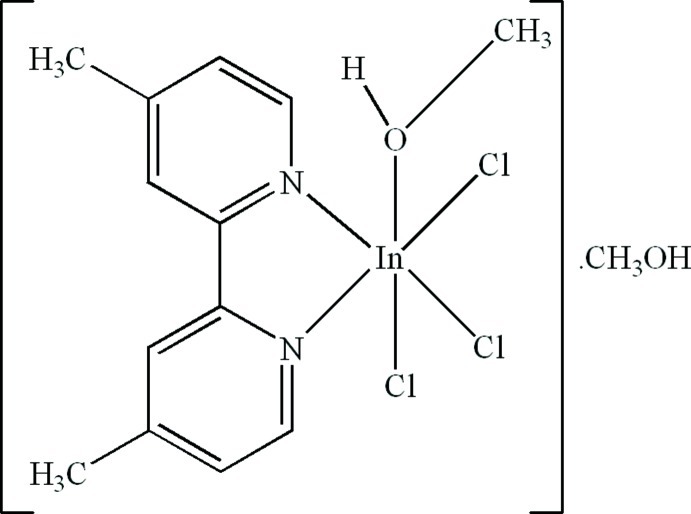


## Experimental

### 

#### Crystal data

[InCl_3_(C_12_H_12_N_2_)(CH_4_O)]·CH_4_O
*M* *_r_* = 469.49
Monoclinic, 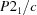

*a* = 12.0318 (6) Å
*b* = 10.3751 (4) Å
*c* = 15.2626 (7) Å
β = 91.981 (4)°
*V* = 1904.11 (15) Å^3^
*Z* = 4
Mo *K*α radiation
μ = 1.67 mm^−1^
*T* = 298 K
0.30 × 0.25 × 0.23 mm

#### Data collection

Bruker APEXII CCD diffractometer
Absorption correction: multi-scan (*SADABS*; Bruker, 2001 ▶) *T*_min_ = 0.621, *T*_max_ = 0.699
11231 measured reflections
3747 independent reflections
3200 reflections with *I* > 2σ(*I*)
*R*_int_ = 0.064

#### Refinement

*R*[*F* ^2^ > 2σ(*F* ^2^)] = 0.040
*wR*(*F* ^2^) = 0.102
*S* = 1.05
3747 reflections
205 parameters
H atoms treated by a mixture of independent and constrained refinement
Δρ_max_ = 1.43 e Å^−3^
Δρ_min_ = −0.76 e Å^−3^

### 

Data collection: *APEX2* (Bruker, 2007 ▶); cell refinement: *SAINT* (Bruker, 2007 ▶); data reduction: *SAINT*; program(s) used to solve structure: *SHELXS97* (Sheldrick, 2008) ▶; program(s) used to refine structure: *SHELXL97* (Sheldrick, 2008) ▶; molecular graphics: *ORTEP-3* (Farrugia, 1997 ▶) and *Mercury* (Macrae *et al.*, 2006 ▶); software used to prepare material for publication: *SHELXTL* (Sheldrick, 2008) ▶.

## Supplementary Material

Crystal structure: contains datablock(s) I, global. DOI: 10.1107/S1600536812035490/hy2578sup1.cif

Structure factors: contains datablock(s) I. DOI: 10.1107/S1600536812035490/hy2578Isup2.hkl

Additional supplementary materials:  crystallographic information; 3D view; checkCIF report

## Figures and Tables

**Table 1:** Hydrogen-bond geometry (Å, °)

*D*—H⋯*A*	*D*—H	H⋯*A*	*D*⋯*A*	*D*—H⋯*A*
O1—H1*B*⋯O2	0.85 (6)	1.83 (6)	2.648 (6)	161 (6)
O2—H2*B*⋯Cl3^i^	0.82	2.77	3.462 (5)	143
C1—H1⋯Cl2	0.93	2.76	3.408 (4)	128
C2—H2⋯Cl1^ii^	0.93	2.77	3.681 (4)	167
C12—H12⋯Cl3	0.93	2.78	3.411 (4)	126

Symmetry codes: (i) 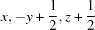; (ii) 
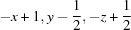
.

**Fig. 1.**
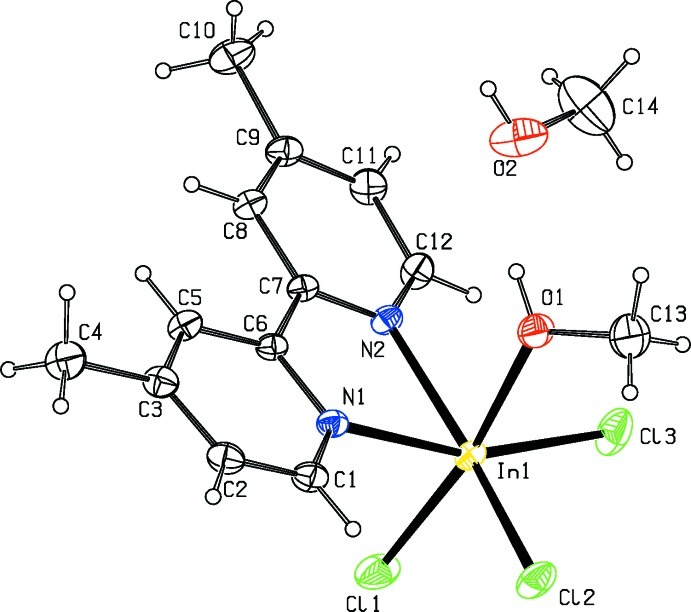
The molecular structure of the title compound. Displacement ellipsoids are drawn at the 30% probability level.

**Fig. 2.**
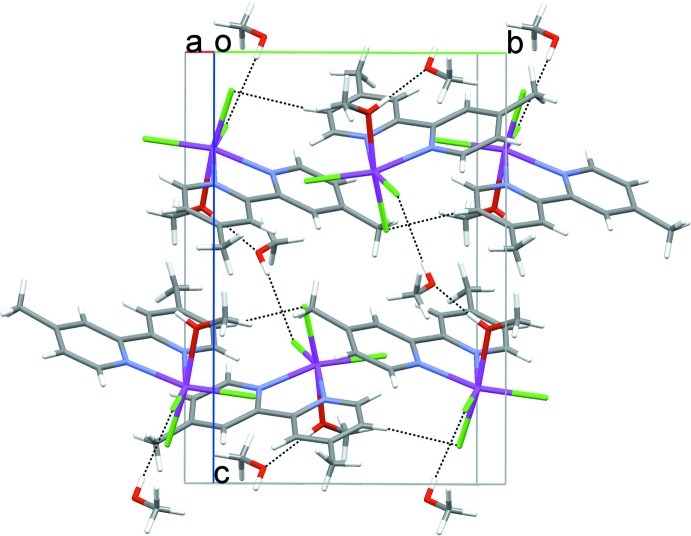
A packing diagram of the title compound. Hydrogen bonds are shown as dashed lines.

## supplementary crystallographic information

### Comment

Several In(III) complexes with a formula [In(*L*_1_)Cl_3_(*L*_2_)] (*L*_1_ = an N,N'-chelating ligand, *L*_2_ = DMSO, H_2_O, MeOH and EtOH), such as [In(bipy)Cl_3_(H_2_O)], (II), [In(bipy)Cl_3_(EtOH)], (III), [In(bipy)Cl_3_(H_2_O)].H_2_O, (IV) (Malyarick *et al.*, 1992), [In(phen)Cl_3_(DMSO)], (V) (Dong *et al.*, 1987), [In(phen)Cl_3_(H_2_O)], (VI), [In(phen)Cl_3_(EtOH)].EtOH, (VII) (Ilyuhin & Malyarick, 1994), [In(4,4'-dmbipy)Cl_3_(DMSO)], (IIX) (Ahmadi *et al.*, 2008), [In(5,5'-dmbipy)Cl_3_(MeOH)], (IX) (Kalateh, Ahmadi *et al.*, 2008), [In(4bt)Cl_3_(MeOH)], (X), and [In(4bt)Cl_3_(DMSO)], (XI) (Abedi *et al.*, 2012) (bipy = 2,2'-bipyridine, phen = 1,10-phenanthroline, DMSO = dimethyl sulfoxide, 4,4'-dmbipy = 4,4'-dimethyl-2,2'-bipyridine, 5,5'-dmbipy = 5,5'-dimethyl-2,2'-bipyridine, 4bt = 4,4'-bithiazole), have been synthesized and characterized by single-crystal X-ray diffraction methods. 4,4'-Dmbipy is a good bidentate ligand, and numerous complexes with 4,4'-dmbipy have been prepared, such as that of [Hg(4,4'-dmbipy)I_2_], (XII) (Yousefi *et al.*, 2008), [Hg(4,4'-dmbipy)Br_2_], (XIII) (Kalateh, Ebadi *et al.*, 2008), [Fe(4,4'-dmbipy)Cl_3_(DMSO)], (XIV) (Amani *et al.*, 2009), [Pt(4,4'-dmbipy)Cl_4_], (XV) (Hojjat Kashani *et al.*, 2008), [Cd(4,4'-dmbipy)I_2_(DMSO)], (XVI) (Kalateh *et al.*, 2010), [Zn(4,4'-dmbipy)Br_2_], (XVII) (Alizadeh *et al.*, 2010), [Zn(4,4'-dmbipy)(H_2_O)(NO_3_)_2_], (XVIII) (Shirvan & Haydari Dezfuli, 2011), and [Cd(4,4'-dmbipy)Br_2_(DMSO)], (XIX) (Shirvan & Haydari Dezfuli, 2012). We report herein the synthesis and crystal structure of the title compound, (I).

In the title compound (Fig. 1), the In^III^ atom is six-coordinated in a distorted octahedral geometry by two N atoms from a chelating 4,4'-dmbipy ligand, one O atom from a methanol molecule and three Cl atoms. There is also one solvent methanol molecule in the asymmetric unit. The In—Cl, In—N and In—O bond lengths and angles are within normal range. In the crystal, intermolecular O—H···O and O—H···Cl hydrogen bonds link the complex and solvent methanol molecules (Fig. 2, Table 1). Intramolecular C—H···Cl hydrogen bonds are present in the complex.

### Experimental

For the preparation of the title compound, a solution of 4,4'-dmbipy (0.30 g, 1.65 mmol) in methanol (20 ml) was added to a solution of InCl_3_.4H_2_O (0.48 g, 1.65 mmol) in methanol (20 ml). The resulting colorless solution was stirred for 10 min at room temperature and then it was left to evaporate slowly at room temperature. After six days, colorless block crystals of the title compound were isolated (yield: 0.62 g, 80.0%).

### Refinement

H atoms bonded to C atoms and O2 atom were positioned geometrically and refined as riding atoms, with C—H = 0.93 (aromatic) and 0.96 (CH_3_) and O—H = 0.82 Å and with *U*_iso_(H) = 1.2(1.5 for hydroxyl)*U*_eq_(C, O). H atom bonded to O1 atom was located from a difference Fourier map and refined isotropically. The highest residual electron density was found at 0.86 Å from In1 atom and the deepest hole at 0.91 Å from In1 atom.

### Crystal data

**Table:**

[InCl_3_(C_12_H_12_N_2_)(CH_4_O)]·CH_4_O	*F*(000) = 936
*M**_r_* = 469.49	*D*_x_ = 1.638 Mg m^−^^3^
Monoclinic, *P*2_1_/*c*	Mo *K*α radiation, λ = 0.71073 Å
Hall symbol: -P 2ybc	Cell parameters from 11231 reflections
*a* = 12.0318 (6) Å	θ = 1.7–26.0°
*b* = 10.3751 (4) Å	µ = 1.67 mm^−^^1^
*c* = 15.2626 (7) Å	*T* = 298 K
β = 91.981 (4)°	Block, colorless
*V* = 1904.11 (15) Å^3^	0.30 × 0.25 × 0.23 mm
*Z* = 4	

### Data collection

**Table:**

Bruker APEXII CCD diffractometer	3747 independent reflections
Radiation source: fine-focus sealed tube	3200 reflections with *I* > 2σ(*I*)
Graphite monochromator	*R*_int_ = 0.064
φ and ω scans	θ_max_ = 26.0°, θ_min_ = 1.7°
Absorption correction: multi-scan (*SADABS*; Bruker, 2001)	*h* = −14→14
*T*_min_ = 0.621, *T*_max_ = 0.699	*k* = −12→12
11231 measured reflections	*l* = −18→18

### Refinement

**Table:**

Refinement on *F*^2^	Primary atom site location: structure-invariant direct methods
Least-squares matrix: full	Secondary atom site location: difference Fourier map
*R*[*F*^2^ > 2σ(*F*^2^)] = 0.040	Hydrogen site location: inferred from neighbouring sites
*wR*(*F*^2^) = 0.102	H atoms treated by a mixture of independent and constrained refinement
*S* = 1.05	*w* = 1/[σ^2^(*F*_o_^2^) + (0.0645*P*)^2^ + 0.029*P*] where *P* = (*F*_o_^2^ + 2*F*_c_^2^)/3
3747 reflections	(Δ/σ)_max_ = 0.009
205 parameters	Δρ_max_ = 1.43 e Å^−^^3^
0 restraints	Δρ_min_ = −0.76 e Å^−^^3^

### Special details

**Table:**

Geometry. All e.s.d.'s (except the e.s.d. in the dihedral angle between two l.s. planes) are estimated using the full covariance matrix. The cell e.s.d.'s are taken into account individually in the estimation of e.s.d.'s in distances, angles and torsion angles; correlations between e.s.d.'s in cell parameters are only used when they are defined by crystal symmetry. An approximate (isotropic) treatment of cell e.s.d.'s is used for estimating e.s.d.'s involving l.s. planes.
Refinement. Refinement of *F*^2^ against ALL reflections. The weighted *R*-factor *wR* and goodness of fit *S* are based on *F*^2^, conventional *R*-factors *R* are based on *F*, with *F* set to zero for negative *F*^2^. The threshold expression of *F*^2^ > σ(*F*^2^) is used only for calculating *R*-factors(gt) *etc*. and is not relevant to the choice of reflections for refinement. *R*-factors based on *F*^2^ are statistically about twice as large as those based on *F*, and *R*- factors based on ALL data will be even larger.

### Fractional atomic coordinates and isotropic or equivalent isotropic displacement parameters (Å^2^)

**Table:**

	*x*	*y*	*z*	*U*_iso_*/*U*_eq_	
In1	0.760984 (19)	0.07129 (2)	0.223229 (16)	0.03869 (11)	
C1	0.5479 (3)	−0.0469 (4)	0.3156 (3)	0.0454 (9)	
H1	0.5727	−0.1222	0.2897	0.054*	
C2	0.4532 (3)	−0.0517 (4)	0.3638 (3)	0.0484 (9)	
H2	0.4158	−0.1293	0.3705	0.058*	
C3	0.4143 (3)	0.0591 (4)	0.4021 (3)	0.0459 (9)	
C4	0.3089 (4)	0.0606 (5)	0.4526 (3)	0.0641 (12)	
H4A	0.2564	0.1185	0.4248	0.077*	
H4B	0.3253	0.0889	0.5115	0.077*	
H4C	0.2779	−0.0246	0.4537	0.077*	
C5	0.4753 (3)	0.1719 (4)	0.3912 (2)	0.0440 (8)	
H5	0.4517	0.2485	0.4162	0.053*	
C6	0.5711 (3)	0.1703 (3)	0.3433 (2)	0.0350 (7)	
C7	0.6408 (3)	0.2864 (3)	0.3311 (2)	0.0344 (7)	
C8	0.6183 (3)	0.4038 (3)	0.3701 (2)	0.0407 (8)	
H8	0.5564	0.4125	0.4044	0.049*	
C9	0.6881 (3)	0.5084 (3)	0.3578 (2)	0.0456 (8)	
C10	0.6694 (4)	0.6323 (4)	0.4057 (3)	0.0684 (13)	
H10A	0.6741	0.6169	0.4678	0.082*	
H10B	0.5971	0.6656	0.3897	0.082*	
H10C	0.7252	0.6938	0.3904	0.082*	
C11	0.7760 (3)	0.4922 (4)	0.3035 (3)	0.0518 (9)	
H11	0.8233	0.5608	0.2925	0.062*	
C12	0.7935 (3)	0.3735 (4)	0.2654 (3)	0.0494 (9)	
H12	0.8525	0.3642	0.2281	0.059*	
C13	0.9285 (5)	−0.0471 (6)	0.3806 (5)	0.094 (2)	
H13A	0.9916	−0.0296	0.3457	0.113*	
H13B	0.9018	−0.1327	0.3684	0.113*	
H13C	0.9500	−0.0404	0.4416	0.113*	
C14	0.9904 (6)	0.3073 (8)	0.4407 (5)	0.121 (3)	
H14A	0.9729	0.3965	0.4312	0.182*	
H14B	1.0182	0.2709	0.3880	0.182*	
H14C	1.0459	0.2998	0.4871	0.182*	
N1	0.6052 (2)	0.0611 (3)	0.3047 (2)	0.0380 (6)	
N2	0.7290 (2)	0.2717 (3)	0.28000 (18)	0.0381 (6)	
O1	0.8436 (3)	0.0429 (3)	0.3603 (2)	0.0572 (8)	
H1B	0.844 (5)	0.109 (6)	0.393 (4)	0.083 (18)*	
O2	0.8937 (4)	0.2405 (5)	0.4641 (3)	0.1071 (15)	
H2B	0.8716	0.2694	0.5104	0.161*	
Cl1	0.65354 (13)	0.12400 (14)	0.08916 (7)	0.0776 (4)	
Cl2	0.76643 (9)	−0.15957 (10)	0.20171 (9)	0.0639 (3)	
Cl3	0.94145 (10)	0.13256 (13)	0.17180 (10)	0.0777 (4)	

### Atomic displacement parameters (Å^2^)

**Table:**

	*U*^11^	*U*^22^	*U*^33^	*U*^12^	*U*^13^	*U*^23^
In1	0.04584 (16)	0.03230 (16)	0.03823 (16)	0.00351 (10)	0.00593 (10)	−0.00244 (10)
C1	0.055 (2)	0.0314 (18)	0.050 (2)	−0.0074 (15)	−0.0027 (17)	−0.0017 (15)
C2	0.053 (2)	0.043 (2)	0.049 (2)	−0.0145 (16)	0.0001 (17)	0.0010 (17)
C3	0.0499 (19)	0.050 (2)	0.0383 (19)	−0.0082 (16)	0.0037 (15)	0.0011 (16)
C4	0.065 (3)	0.066 (3)	0.063 (3)	−0.015 (2)	0.019 (2)	−0.001 (2)
C5	0.0528 (19)	0.0374 (19)	0.0423 (19)	−0.0021 (15)	0.0097 (15)	−0.0041 (16)
C6	0.0446 (16)	0.0293 (16)	0.0310 (16)	−0.0010 (13)	−0.0014 (13)	0.0003 (13)
C7	0.0441 (16)	0.0294 (16)	0.0296 (15)	−0.0001 (13)	0.0005 (12)	0.0001 (13)
C8	0.0510 (18)	0.0347 (18)	0.0368 (18)	−0.0004 (15)	0.0062 (15)	−0.0010 (15)
C9	0.062 (2)	0.0288 (18)	0.046 (2)	−0.0016 (16)	−0.0024 (17)	0.0004 (16)
C10	0.094 (3)	0.034 (2)	0.079 (3)	−0.007 (2)	0.010 (3)	−0.010 (2)
C11	0.055 (2)	0.033 (2)	0.068 (3)	−0.0083 (17)	0.0037 (19)	0.0033 (19)
C12	0.0480 (19)	0.040 (2)	0.061 (2)	−0.0027 (16)	0.0139 (18)	0.0007 (18)
C13	0.095 (4)	0.090 (4)	0.095 (4)	0.046 (3)	−0.033 (3)	−0.009 (3)
C14	0.106 (5)	0.128 (7)	0.128 (6)	−0.039 (5)	−0.015 (4)	0.048 (5)
N1	0.0435 (14)	0.0322 (15)	0.0381 (15)	−0.0012 (11)	−0.0018 (12)	−0.0026 (12)
N2	0.0452 (15)	0.0282 (15)	0.0411 (15)	−0.0007 (11)	0.0057 (12)	−0.0019 (12)
O1	0.0683 (18)	0.0521 (17)	0.0504 (17)	0.0180 (14)	−0.0104 (14)	−0.0038 (14)
O2	0.144 (4)	0.099 (3)	0.078 (3)	−0.015 (3)	0.008 (3)	−0.027 (3)
Cl1	0.1109 (9)	0.0774 (8)	0.0431 (6)	0.0324 (7)	−0.0173 (6)	−0.0093 (6)
Cl2	0.0772 (7)	0.0346 (5)	0.0808 (7)	0.0035 (4)	0.0164 (6)	−0.0134 (5)
Cl3	0.0636 (6)	0.0626 (7)	0.1095 (10)	−0.0022 (5)	0.0415 (7)	−0.0084 (7)

### Geometric parameters (Å, º)

**Table:**

In1—Cl1	2.4443 (13)	C9—C10	1.500 (5)
In1—Cl2	2.4188 (11)	C9—C11	1.376 (5)
In1—Cl3	2.4192 (13)	C11—C12	1.381 (6)
In1—O1	2.304 (3)	C1—H1	0.9300
In1—N1	2.287 (3)	C2—H2	0.9300
In1—N2	2.290 (3)	C4—H4B	0.9600
O1—C13	1.411 (7)	C4—H4C	0.9600
O1—H1B	0.85 (6)	C4—H4A	0.9600
O2—C14	1.411 (9)	C5—H5	0.9300
O2—H2B	0.8200	C8—H8	0.9300
N1—C1	1.329 (5)	C10—H10A	0.9600
N1—C6	1.348 (4)	C10—H10C	0.9600
N2—C7	1.347 (4)	C10—H10B	0.9600
N2—C12	1.334 (5)	C11—H11	0.9300
C1—C2	1.378 (6)	C12—H12	0.9300
C2—C3	1.379 (6)	C13—H13C	0.9600
C3—C4	1.507 (6)	C13—H13A	0.9600
C3—C5	1.394 (6)	C13—H13B	0.9600
C5—C6	1.386 (5)	C14—H14A	0.9600
C6—C7	1.483 (5)	C14—H14B	0.9600
C7—C8	1.387 (4)	C14—H14C	0.9600
C8—C9	1.389 (5)		
			
Cl1···Cl2	3.6456 (18)	N2···H1B	2.75 (6)
Cl1···Cl3	3.646 (2)	C1···C7^ii^	3.579 (5)
Cl1···N1	3.423 (3)	C1···C8^ii^	3.450 (5)
Cl1···N2	3.387 (3)	C2···C7^ii^	3.566 (5)
Cl1···C8^i^	3.368 (3)	C2···C12^ii^	3.591 (6)
Cl2···O1	3.313 (3)	C5···Cl2^iii^	3.639 (4)
Cl2···N1	3.419 (3)	C7···C1^iii^	3.579 (5)
Cl2···C13	3.500 (7)	C7···C2^iii^	3.566 (5)
Cl2···C5^ii^	3.639 (4)	C8···C1^iii^	3.450 (5)
Cl2···Cl1	3.6456 (18)	C8···Cl1^v^	3.368 (3)
Cl2···C1	3.408 (4)	C12···C2^iii^	3.591 (6)
Cl3···Cl1	3.646 (2)	C12···C14	3.578 (9)
Cl3···C12	3.411 (4)	C13···O2	3.277 (8)
Cl3···O1	3.281 (3)	C14···Cl3^v^	3.650 (8)
Cl3···N2	3.411 (3)	C14···C12	3.578 (9)
Cl3···C14^i^	3.650 (8)	C1···H4B^vi^	3.0300
Cl3···O2^i^	3.462 (5)	C5···H8	2.6900
Cl1···H10A^i^	3.1300	C8···H5	2.6800
Cl1···H2B^i^	3.1300	C14···H1B	2.79 (6)
Cl1···H8^i^	3.0400	H1···Cl2	2.7600
Cl1···H2^iii^	2.7700	H1B···O2	1.83 (6)
Cl1···H10B^ii^	3.0700	H1B···C14	2.79 (6)
Cl2···H14B^iv^	3.0600	H1B···H2B	2.4600
Cl2···H13B	2.9900	H2···H4C	2.3900
Cl2···H4A^ii^	3.0100	H2···Cl1^ii^	2.7700
Cl2···H1	2.7600	H2B···H1B	2.4600
Cl3···H14A^iv^	3.1100	H2B···Cl1^v^	3.1300
Cl3···H12	2.7800	H2B···Cl3^v^	2.7700
Cl3···H11^iv^	2.9600	H4A···Cl2^iii^	3.0100
Cl3···H2B^i^	2.7700	H4B···C1^vi^	3.0300
O1···Cl2	3.313 (3)	H4C···H2	2.3900
O1···Cl3	3.281 (3)	H5···H8	2.1300
O1···O2	2.648 (6)	H5···C8	2.6800
O1···N1	2.969 (4)	H8···H5	2.1300
O1···N2	2.987 (4)	H8···C5	2.6900
O2···Cl3^v^	3.462 (5)	H8···Cl1^v^	3.0400
O2···O1	2.648 (6)	H10A···Cl1^v^	3.1300
O2···C13	3.277 (8)	H10B···Cl1^iii^	3.0700
O2···H1B	1.83 (6)	H10C···H11	2.3800
N1···Cl2	3.419 (3)	H11···Cl3^vii^	2.9600
N1···O1	2.969 (4)	H11···H10C	2.3800
N1···N2	2.678 (4)	H12···H13A^vii^	2.4800
N1···Cl1	3.423 (3)	H12···Cl3	2.7800
N1···C7	2.408 (4)	H13A···H12^iv^	2.4800
N2···N1	2.678 (4)	H13B···Cl2	2.9900
N2···Cl1	3.387 (3)	H13C···H13C^viii^	2.2800
N2···Cl3	3.411 (3)	H14A···Cl3^vii^	3.1100
N2···C6	2.403 (4)	H14B···Cl2^vii^	3.0600
N2···O1	2.987 (4)		
			
Cl1—In1—Cl2	97.12 (5)	C10—C9—C11	122.0 (3)
Cl1—In1—Cl3	97.14 (5)	C9—C11—C12	119.6 (4)
Cl1—In1—O1	171.08 (9)	N2—C12—C11	122.5 (4)
Cl1—In1—N1	92.63 (8)	C2—C1—H1	119.00
Cl1—In1—N2	91.30 (8)	N1—C1—H1	119.00
Cl2—In1—Cl3	100.80 (4)	C3—C2—H2	120.00
Cl2—In1—O1	89.07 (8)	C1—C2—H2	120.00
Cl2—In1—N1	93.13 (8)	C3—C4—H4A	109.00
Cl2—In1—N2	162.96 (8)	C3—C4—H4C	110.00
Cl3—In1—O1	87.96 (9)	H4A—C4—H4B	109.00
Cl3—In1—N1	161.82 (8)	C3—C4—H4B	109.00
Cl3—In1—N2	92.79 (7)	H4B—C4—H4C	109.00
O1—In1—N1	80.58 (11)	H4A—C4—H4C	110.00
O1—In1—N2	81.11 (10)	C3—C5—H5	120.00
N1—In1—N2	71.62 (10)	C6—C5—H5	120.00
In1—O1—C13	124.8 (4)	C9—C8—H8	120.00
C13—O1—H1B	115 (4)	C7—C8—H8	120.00
In1—O1—H1B	115 (4)	C9—C10—H10A	109.00
C14—O2—H2B	109.00	C9—C10—H10C	109.00
In1—N1—C1	123.1 (3)	H10A—C10—H10B	109.00
In1—N1—C6	117.8 (2)	H10A—C10—H10C	109.00
C1—N1—C6	119.2 (3)	H10B—C10—H10C	110.00
C7—N2—C12	118.8 (3)	C9—C10—H10B	110.00
In1—N2—C7	117.9 (2)	C12—C11—H11	120.00
In1—N2—C12	123.3 (2)	C9—C11—H11	120.00
N1—C1—C2	122.6 (4)	N2—C12—H12	119.00
C1—C2—C3	119.7 (4)	C11—C12—H12	119.00
C2—C3—C4	122.0 (4)	O1—C13—H13B	110.00
C4—C3—C5	120.5 (4)	O1—C13—H13C	110.00
C2—C3—C5	117.5 (4)	O1—C13—H13A	109.00
C3—C5—C6	120.2 (4)	H13A—C13—H13C	109.00
N1—C6—C7	116.5 (3)	H13B—C13—H13C	109.00
N1—C6—C5	120.8 (3)	H13A—C13—H13B	109.00
C5—C6—C7	122.7 (3)	O2—C14—H14A	109.00
N2—C7—C6	116.2 (3)	O2—C14—H14B	109.00
N2—C7—C8	121.2 (3)	O2—C14—H14C	109.00
C6—C7—C8	122.7 (3)	H14A—C14—H14B	110.00
C7—C8—C9	120.0 (3)	H14A—C14—H14C	109.00
C8—C9—C11	117.8 (3)	H14B—C14—H14C	109.00
C8—C9—C10	120.2 (3)		
			
Cl2—In1—O1—C13	38.9 (4)	C1—N1—C6—C7	178.0 (3)
Cl3—In1—O1—C13	−62.0 (4)	In1—N2—C12—C11	−176.9 (3)
N1—In1—O1—C13	132.2 (4)	C7—N2—C12—C11	2.7 (6)
N2—In1—O1—C13	−155.1 (4)	In1—N2—C7—C8	177.9 (2)
Cl1—In1—N1—C1	90.5 (3)	In1—N2—C7—C6	−1.6 (4)
Cl1—In1—N1—C6	−89.8 (2)	C12—N2—C7—C6	178.8 (3)
Cl2—In1—N1—C1	−6.8 (3)	C12—N2—C7—C8	−1.7 (5)
Cl2—In1—N1—C6	173.0 (2)	N1—C1—C2—C3	0.6 (7)
O1—In1—N1—C1	−95.4 (3)	C1—C2—C3—C5	−1.2 (6)
O1—In1—N1—C6	84.4 (2)	C1—C2—C3—C4	177.6 (4)
N2—In1—N1—C1	−179.0 (3)	C2—C3—C5—C6	0.1 (6)
N2—In1—N1—C6	0.7 (2)	C4—C3—C5—C6	−178.8 (4)
Cl1—In1—N2—C7	92.9 (2)	C3—C5—C6—C7	−178.6 (3)
Cl1—In1—N2—C12	−87.6 (3)	C3—C5—C6—N1	1.8 (5)
Cl3—In1—N2—C7	−169.9 (2)	C5—C6—C7—N2	−177.4 (3)
Cl3—In1—N2—C12	9.7 (3)	C5—C6—C7—C8	3.1 (5)
O1—In1—N2—C7	−82.4 (2)	N1—C6—C7—N2	2.3 (4)
O1—In1—N2—C12	97.1 (3)	N1—C6—C7—C8	−177.2 (3)
N1—In1—N2—C7	0.5 (2)	C6—C7—C8—C9	178.6 (3)
N1—In1—N2—C12	−179.9 (3)	N2—C7—C8—C9	−0.9 (5)
In1—N1—C1—C2	−179.1 (3)	C7—C8—C9—C11	2.6 (5)
C6—N1—C1—C2	1.2 (6)	C7—C8—C9—C10	−175.0 (3)
In1—N1—C6—C5	177.9 (2)	C8—C9—C11—C12	−1.7 (6)
C1—N1—C6—C5	−2.4 (5)	C10—C9—C11—C12	175.9 (4)
In1—N1—C6—C7	−1.8 (4)	C9—C11—C12—N2	−1.0 (6)

Symmetry codes: (i) *x*, −*y*+1/2, *z*−1/2; (ii) −*x*+1, *y*−1/2, −*z*+1/2; (iii) −*x*+1, *y*+1/2, −*z*+1/2; (iv) −*x*+2, *y*−1/2, −*z*+1/2; (v) *x*, −*y*+1/2, *z*+1/2; (vi) −*x*+1, −*y*, −*z*+1; (vii) −*x*+2, *y*+1/2, −*z*+1/2; (viii) −*x*+2, −*y*, −*z*+1.

### Hydrogen-bond geometry (Å, º)

**Table:**

*D*—H···*A*	*D*—H	H···*A*	*D*···*A*	*D*—H···*A*
O1—H1*B*···O2	0.85 (6)	1.83 (6)	2.648 (6)	161 (6)
O2—H2*B*···Cl3^v^	0.82	2.77	3.462 (5)	143
C1—H1···Cl2	0.93	2.76	3.408 (4)	128
C2—H2···Cl1^ii^	0.93	2.77	3.681 (4)	167
C12—H12···Cl3	0.93	2.78	3.411 (4)	126

Symmetry codes: (ii) −*x*+1, *y*−1/2, −*z*+1/2; (v) *x*, −*y*+1/2, *z*+1/2.

## References

[bb1] Abedi, A., Safari, N., Amani, V. & Khavasi, H. R. (2012). *J. Coord. Chem.* **65**, 325–338.

[bb2] Ahmadi, R., Kalateh, K., Abedi, A., Amani, V. & Khavasi, H. R. (2008). *Acta Cryst.* E**64**, m1306–m1307. 10.1107/S1600536808029553 PMC2959453 21201045

[bb3] Alizadeh, R., Mohammadi Eshlaghi, P. & Amani, V. (2010). *Acta Cryst.* E**66**, m996. 10.1107/S1600536810028692 PMC3007259 21588215

[bb4] Amani, V., Safari, N., Notash, B. & Khavasi, H. R. (2009). *J. Coord. Chem.* **62**, 1939–1950.

[bb5] Bruker (2001). *SADABS* Bruker AXS Inc., Madison, Wisconsin, USA.

[bb6] Bruker (2007). *APEX2* and *SAINT* Bruker AXS Inc., Madison, Wisconsin, USA.

[bb7] Dong, N., Hang, N.-D., Dong, Z.-C. & Hu, S.-Z. (1987). *Jiegou Huaxue (Chin. J. Struct. Chem.)*, **6**, 145–149.

[bb8] Farrugia, L. J. (1997). *J. Appl. Cryst.* **30**, 565.

[bb9] Hojjat Kashani, L., Amani, V., Yousefi, M. & Khavasi, H. R. (2008). *Acta Cryst.* E**64**, m905–m906. 10.1107/S1600536808016796 PMC2961848 21202769

[bb10] Ilyuhin, A. B. & Malyarick, M. A. (1994). *Kristallografiya*, **39**, 439–443.

[bb11] Kalateh, K., Ahmadi, R. & Amani, V. (2010). *Acta Cryst.* E**66**, m512. 10.1107/S1600536810012572 PMC2979279 21579009

[bb12] Kalateh, K., Ahmadi, R., Ebadi, A., Amani, V. & Khavasi, H. R. (2008). *Acta Cryst.* E**64**, m1353–m1354. 10.1107/S160053680803119X PMC2959790 21580816

[bb13] Kalateh, K., Ebadi, A., Ahmadi, R., Amani, V. & Khavasi, H. R. (2008). *Acta Cryst.* E**64**, m1397–m1398. 10.1107/S1600536808032510 PMC2959555 21580848

[bb14] Macrae, C. F., Edgington, P. R., McCabe, P., Pidcock, E., Shields, G. P., Taylor, R., Towler, M. & van de Streek, J. (2006). *J. Appl. Cryst.* **39**, 453–457.

[bb15] Malyarick, M. A., Petrosyants, S. P. & Ilyuhin, A. B. (1992). *Polyhedron*, **11**, 1067–1073.

[bb19] Sheldrick, G. M. (2008). *Acta Cryst* A**64**, 112–122. 10.1107/S0108767307043930 18156677

[bb16] Shirvan, S. A. & Haydari Dezfuli, S. (2011). *Acta Cryst.* E**67**, m1866–m1867. 10.1107/S1600536811050227 PMC3238756 22199633

[bb17] Shirvan, S. A. & Haydari Dezfuli, S. (2012). *Acta Cryst.* E**68**, m1006–m1007. 10.1107/S1600536812028553 PMC3393245 22807705

[bb18] Yousefi, M., Tadayon Pour, N., Amani, V. & Khavasi, H. R. (2008). *Acta Cryst.* E**64**, m1259. 10.1107/S1600536808028791 PMC2959485 21201014

